# Managing early complications in total hip arthroplasty: the safety of immediate revision

**DOI:** 10.1186/s10195-023-00719-1

**Published:** 2023-07-31

**Authors:** Jules Descamps, Victoria Teissier, Wilfrid Graff, Antoine Mouton, Pierre-Alban Bouché, Simon Marmor

**Affiliations:** 1 Bone-and-Joint Infections Referral Center, Groupe Hospitalier Diaconnesses Croix Saint-Simon, 125 Rue d’Avron, 75020 Paris, France; 2 Orthopedic Surgery Departement, Groupe Hospitalier Diaconnesses Croix Saint-Simon, 125 Rue d’Avron, 75020 Paris, France

**Keywords:** Leg length discrepancy, Total hip arthroplasty, Immediate revision, Infection, Complication rates

## Abstract

**Purpose:**

Immediate revision refers to a reoperation that involves resetting, draping, and exchanging the implant, after wound closure in total hip arthroplasty. The purpose of this study is to investigate the impact of immediate revision after total hip arthroplasty on subsequent infection and complication rates.

**Methods:**

A total of 14,076 primary total hip arthroplasties performed between 2010 and 2020 were identified in our institutional database, of which 42 underwent immediate revision. Infection rates were determined 2 years after the index arthroplasty. The cause and type of revision, duration of primary and revision surgeries, National Nosocomial Infections Surveillance score, implant type, changes in implants, complications, and preoperative and intraoperative antibiotic prophylaxis were all determined.

**Results:**

No infections were observed within 2 years after the index arthroplasty. Leg length discrepancy (88%, *n* = 37) and dislocation (7.1%, *n* = 3) were the main causes of immediate revision. In most cases of discrepancy, the limb was clinically and radiologically longer before the immediate revision. The mean operative time was 48 ± 14 min for the primary procedure and 23.6 ± 9 min for the revision. The time between the first incision and last skin closure ranged from 1 to 3 h. None of the patients were extubated between the two procedures. Two patients had a National Nosocomial Infections Surveillance score of 2, 13 had a score of 1, and 27 had a score of 0.

**Conclusion:**

Immediate revision is safe for correcting clinical and radiological abnormalities, and may not be associated with increased complication or infection rates.

**Study design:**

Retrospective cohort study; level of evidence, 3.

## Introduction

Total hip arthroplasty (THA) is considered “the operation of the century” [1] for patients with symptomatic hip osteoarthritis in whom conservative treatments have failed. However, residual symptoms are experienced by approximately 9.1% of patients, mainly due to leg length discrepancy (LLD) [2], which rarely requires revision. Malpractice litigation is most commonly associated with nerve injury (38.46%), LLD (26.15%), and instability (12.31%) [3, 4].

Intraoperative testing is ineffective at detecting and reducing LLD to < 15 mm [5]. However, an LLD of > 5 mm is likely to be perceived and should not be tolerated [6]. Revision is necessary when symptomatic LLD (hip or back pain, instability, and paresthesia) occurs and the discrepancy exceeds 20 mm [7]. Instability, one of the main reasons for early revision, occurs in approximately 2% of cases in the first week after surgery [8].

Several studies have indicated an increased risk of infection associated with early revisions (i.e., in the year following index surgery) [9–11]. A recent study suggested that early aseptic THA revision was associated with an increased risk of subsequent periprosthetic joint infection (PJI) [9]. Heckmann et al. demonstrated a relationship between the timing of revision and the risk of PJI; revisions performed within 3 months had significantly higher rates of PJI than revisions done after 12 months; the rate of PJI decreased from approximately 12.7% when the revision was performed at < 1 month to 10.6% at 2–3 months and 6.9% at > 12 months [10]. This could be due to early revisions causing a “second hit” during the physiological recovery from the index surgery. Poor soft tissue quality may also increase colonization and infection rates [11].

Given the increased longevity of hip implants [12] and the younger age of patients undergoing THA [13],

it is important to address any initial imperfections or complications, such as leg length discrepancy (LLD) and instability, promptly. Some authors suggest delaying early revision surgery until after the critical 3 month period, but revision can become more complex, especially if the implant has osseointegrated [10]. However, managing early complications immediately after surgery could have implications that are currently not well understood, especially in the context of immediate revision surgery in THA. Currently, there is limited knowledge about the potential risks and benefits associated with immediate revision surgery in THA. This therapeutical option of immediate revision refers to resetting, draping, and exchanging the implant after wound closure. Knowing the risk of immediate revision can help surgeons make informed decisions. The aim of this study is to investigate the association between immediate revision surgery after primary THA and infection. We also assess the rate of complications, and describe early complications that required immediate revision. The hypothesis is that immediate revision surgery does not increase the risk of infection or complications and provides benefits.

## Methods

**Fig. 1:**
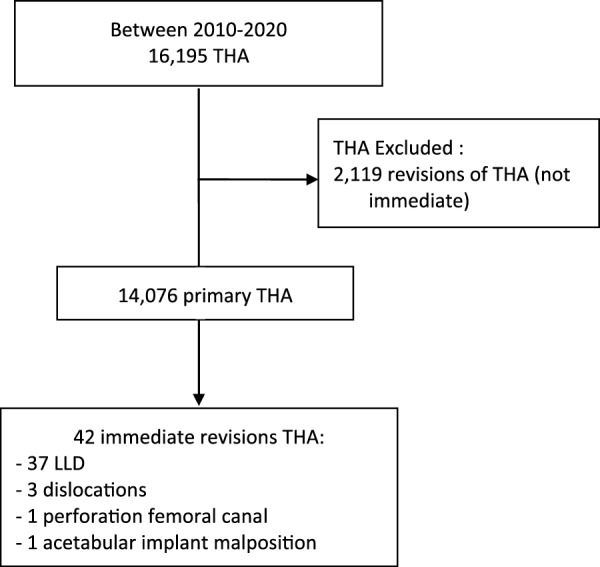
Study flowchart. *THA* total hip arthroplasty, *LLD* leg length discrepancy

This observational study analyzed data from a prospectively collected database of hip arthroplasty. The study adhered to the principles of the Declaration of Helsinki, and was deemed standard by the institutional review board (MR-003). Patients provided consent for the analysis of their clinical and radiologic data. The authors conducted a retrospective review of data from all patients who underwent total hip arthroplasty (THA) between 2010 and 2020, excluding those who had undergone revision hip arthroplasty. To be included as an “immediate revision,” patients had to undergo a primary THA and have a reoperation with an implant exchange performed on the same day as the initial surgery due to early complications [such as leg length discrepancy (LLD) or dislocation]. The decision for immediate revision was made by the senior surgeon. For LLD, the senior surgeon performed a clinical examination of the leg and analyzed the postoperative x-ray. The study analyzed 42 primary THA patients who underwent immediate revision out of the 14,076 eligible THA patients reviewed (Fig. 1).

All primary THA patients underwent surgery via an anterior Hueter approach under general or spinal anesthesia, with a senior surgeon performing the procedure assisted by a resident and a nurse. Preoperative antibiotic prophylaxis was administered intravenously at least 30 min prior to skin incision. Patients were placed on a traction table with pelvic support, feet in laced shoes, and the operated hip was slightly abducted and flexed. The hip was covered with an antimicrobial incision drape containing iodine. The traction table was operated by the nurse in accordance with the surgeon’s instructions. The implant size and position were preoperatively planned using digital 2D templating by the senior surgeon and resident. The implants used were either full hydroxyapatite coating (Meije stems) or cemented stem (Oceane stems, Cirencester, UK) sealed with antibiotic-free cement; the acetabular implant was always cementless (Dynacup, Cirencester, UK), with ceramic delta as a low-friction connection. Wound closure was performed using Monocryl 3/0 sutures or staples. After surgery and prior to patient transfer from the operating room, anteroposterior (AP) radiographs of the pelvis were obtained systematically by a team of trained radiographic manipulators, with the hip kept in 20° internal rotation, and checked for leg length. Digitalized AP radiographs were employed for the analysis and determination of LLD. Measurement of LLD was conducted by the surgeon, using two landmarks: the inferior aspect of the ischial tuberosities and the most prominent medial point of the lesser trochanter, as referenced in the literature [14]. A line was drawn tangential to the lower borders of the ischial tuberosities and the vertical distance to the apex of the lesser trochanter was measured on each side. The difference between these two measurements provided the LLD. These measurements were documented in the surgical report for each patient, supporting the decision for immediate revision when needed. It should be noted that these measurements were taken once by the surgeon due to the clinical immediacy of the decision-making process. The surgeon monitored bed transfer to check the clinical LLD and avoid any abnormal movement of the hip.

### Outcomes

Patient demographics, including age, sex, body mass index (BMI), American Society of Anesthesiologists (ASA) score, smoking status, and comorbidities, were collected. Surgical data were also recorded, including the etiology of the primary THA, the implants and sizes used in the initial surgery, operative time, cause of immediate revision, clinical and radiological LLD, operative time of the revision, and implant change. The patients were followed up for 2 years to monitor subsequent complications, including infections and revisions. The National Nosocomial Infections Surveillance (NNIS) score [15] was calculated using three criteria: an ASA score ≥ 3 (1 point), operation time > 75th percentile (90 min for primary THA) (1 point), and Altemeier classification [16] (always clean operation, 0 points).

The required number of subjects was estimated assuming that the proportion of infections for the reference (infection rate in primary THA) and the expected infection rate from the early revision study would be 0.63% [17] and 6.8% [11], respectively. With a type 1 risk of 5.0%, a type 2 risk of 80.0%, a bilateral test, and a dropout rate estimated at 10.0%, a required sample size of 36 patients was need. Continuous quantitative variables were presented as median and range, while dichotomous variables were presented as the number of events and percentage. Statistical analyses were conducted using R software (version 3.5.0).

## Results

Of 14,076 primary THAs that were performed, 42 (0.3%) required immediate revision. Of 42, 23 were women (54.8%) and 19 were men (45.2%). The median age and BMI were 66.6 years (range, 42–87 years) and 26.4 kg/m^2^ (range, 20.3–33.3 kg/m^2^), respectively. The ASA score was 2 in 61.9% of cases. The median length of stay was 4 days (range, 2–7 days). The patients were followed-up for at least 2 years. All 42 immediate revisions were performed under general anesthesia after the primary THA. The most common indications for immediate revision after primary THA were LLD (*n* = 37, 88%) and dislocation (*n* = 3, 7.1%). One patient required immediate revision for perforation of the femoral canal, and one for stem misalignment and incorrect acetabular implant positioning (not at the bottom of the reamed acetabulum) (Table 1). Of the patients with leg length discrepancy, 86.5% (32/37) had a longer operated leg before revision and 59.5% had a difference of more than 10 mm (range 5–20 mm). All patients were Altemeier grade 0, as defined by primary THA. Eight patients had an ASA score of ≥ 3. Only in seven surgeries (16.7%) did the addition of the two surgery times exceed the 75th percentile of surgery time (90 min for the primary THA). The NNIS was divided into three sections: 2 patients (4.7%) had an NNIS score of 2, 13 (31%) had 1, and 27 (64.3%) had 0.

**Table 1:** Patient demographics

Variables	Total (*n* = 42)
Age [years, median (range)]	66.6 (42–87)
Sex (*n* men, %)	19 (45.2)
Side (*n* left, %)	21 (50.0)
BMI [median (range)]	26.4 (17.7–36.7)
ASA: *n* (%)	
0	1 (2.4)
1	7 (16.7)
2	26 (61.9)
3	8 (19.0)
Comorbidities	
Smoking: *n* (%)	6 (14.3)
Diabetes: *n* (%)	3 (7.1)
Hypertension *n* (%)	17 (40.5)
Heart disease *n* (%)	6 (14.3)
Length of stay in days [median (range)]	4 (2–7)
Etiology of THA *n* (%)	
Osteoarthritis	36 (85.7)
Dysplasia	5 (11.9)
Rapidly destructive osteoarthritis	1 (2.4)
Cause of revision *n* (%)	
LLD	37 (88.1)
Dislocation	3 (7.1)
Perforation femoral canal	1 (2.4)
Acetabular implant positioning	1 (2.4)

*BMI* body mass index, *ASA* American Society of Anesthesiologists, *THA* total hip arthroplasty, *LLD* leg length discrepancy

### Complications

During the 2 year follow-up period, no infections or dislocations were observed in the population who underwent immediate revisions. One patient experienced a trochanteric fracture after a fall 6 weeks after the index surgery, which did not require revision surgery. Another patient died 18 months after surgery due to a fall from a truck. All leg length discrepancies (LLDs) had resolved, with measurements less than 5 mm, except for one patient who still had LLD and was using a compensatory insole at the last follow-up.

Surgical characteristics.

The median operative time was 45 min (range, 30–80 min) and 20 min (range, 11–55 min) for the primary procedure and revision, respectively. The median time between the first incision and last skin closure was 92 min (range, 60–180 min). The median interval between skin closure and the new incision was 29 min (range, 10–100 min). None of the patients were extubated between the two procedures. All patients received antibiotics 30 min preoperatively: cefazolin (38, 90.5%) and vancomycin (4, 9.5%). Only five (12%) patients received intraoperative antibiotic re-injections during the second surgery.

### Characteristics of the implants

**Fig. 2:**
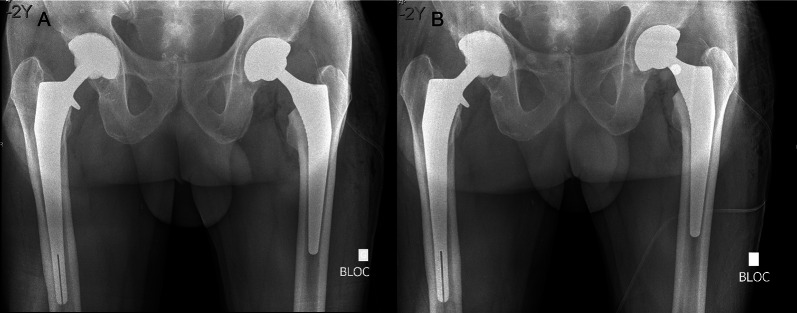
X-ray after postoperative index (**A**) and revision surgery (**B**). Change of two sizes (+4 mm to −4 mm) with an 8 mm LLD. *LLD* leg length discrepancy

**Fig. 3:**
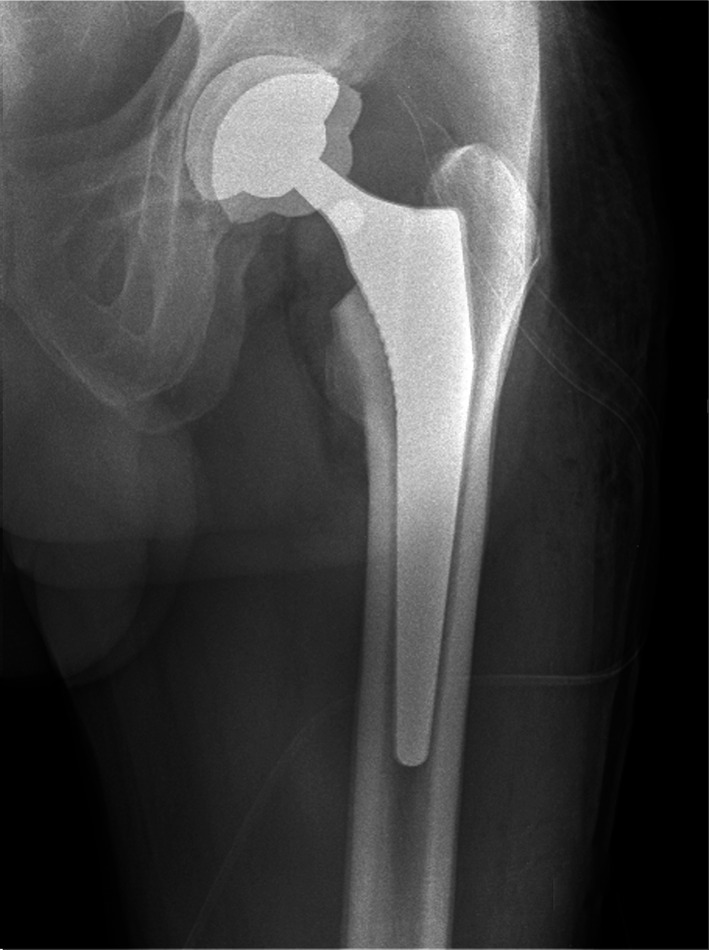
Superposition x-ray femoral stem between index and revision surgery, shortening the leg

The revision surgery consisted of 13 stem revisions, including one in which the same stem was recemented in the femoral canal. Only one acetabular cup was replaced. All the 41 femoral head implants were changed. Nine patients underwent stem and femoral head revisions (Table 2). Figure 2 shows a left postoperative radiograph after the index procedure (Fig. 2A) with an increased femoral offset and excess length and after revision surgery (Fig. 2B) with restored offset and length. Figure 3 shows a reconstructed image superposing the femoral stem and demonstrates the change in offset and length between the two femoral heads, shortening the leg (Fig. 3).

**Table 2:** Revision surgery characteristics

Variables	Total (*n* = 42)
Duration (min)	
Index surgery [median (range)]	45 (30–80)
Revision surgery [median (range)]	20 (11–55)
Interval surgery [median (range)]	27 (8–100)
Total surgery [median (range)]	92.5 (60–195)
New antibiotic injection *n* (%)	5 (12)
Stem revision *n* (%)	13 (30.9)
Smaller (≤ 2 size difference)	1
Smaller (≤ 1 size)	3
Same size	3
Bigger (≥ 1 size)	3
Lateralized	3
Acetabular cup revision *n* (%)	1 (2.4)
Same size	1
Head change *n* (%)	41 (97.6)
Smaller (≤ 2 size difference)	2
Smaller (≤ 1 size)	24
Same size	6
Longer (≥ 1 size)	5
Longer (≥ 2 size difference)	4

*min* minutes

## Discussion

In this study, there was no association between immediate revisions after primary THA and increased complication rates, particularly infection rates, within 2 years of the index procedure. Although dislocation remains an immediate postoperative concern, leg length discrepancy (LLD) is also a main cause of immediate revision. These results could assist surgeons in determining whether immediate revision is necessary, rather than waiting for discomfort at first weight bearing.

Revision surgeries remain a major challenge for surgeons and patients due to the higher 30 day mortality rate [18]. Postoperative complications and the subsequent need for revision also pose a greater economic burden to the healthcare system [19]. Revisions due to preventable causes, such as instability, are expected to increase in the future [20].

Some teams have used intraoperative radiographs [21, 22] or digital radiographic alignment software [23] to avoid implant misplacement or LLD. However, this requires an experienced team with intraoperative radiographs and increases operative time for each primary THA. Intraoperative radiographs may not be precise enough and may not be correlated with postoperative radiographs [24]. Due to the traction table, the authors were unable to take radiographs of both hips to check for LLD. Other teams have used a standard instead of a traction table to intraoperatively control lengths, with some reporting leg length reconstruction as more accurate in the traction table group (0.56 versus 1.78 mm)[25]. However, others have reported that both techniques provided equal results [5], and some have reported that leg length restoration was significantly more accurate without a traction table (2.4 versus 3.7 mm)[26].

According to some authors, revision of THA for LLD may not always be warranted due to the increased risk of complications associated with early revisions [9–11, 20]. Postoperative symptomatic LLD can have a negative impact on patient satisfaction, functional outcomes, and implant survival [27]. This “surgeon-controlled variable” can cause persistent pain, instability, and early failure due to impingement and increased surface wear [28]. Symptomatic LLD can also exacerbate lower back pain, further increasing the economic burden [29].

In our study, the rate of immediate revision after primary THA was 0.3%, which is relatively low. However, immediate revision should still be considered a serious option in clinical practice. Revision surgery, particularly for aseptic reasons, carries the risk of infection, which is the most feared complication. Studies based on insurance registries have reported an increased risk of infection following revision surgery. Goldman et al. [9] analyzed a cohort of 15,357 patients who underwent THA, of whom 211 required aseptic revision within 1 year of the index surgery. They found that aseptic revisions within the first year were associated with an 8- to 13-fold increase in periprosthetic joint infection (PJI) compared with the control group. At 2 years, the PJI rate was 0.2% in the control group, 4.8% in patients who underwent aseptic revision within 90 days [hazard ratio (HR) 8, *p* < 0.001], and 3.2% in patients who underwent aseptic revision between three and 12 months (HR 13, *p* < 0.001), in line with the findings of Quinlan et al.[11]. Out of 5500 primary THA from the Medicare and Humana databases, 550 patients who underwent early aseptic revision (within 1 year of index surgery) were analyzed. They found a significantly increased risk of infection at 1 year in the aseptic revision group when compared with the control group: 5.49% versus 0.91%, OR 5.61, *p* < 0.001 for the Medicare registry base, and 7.21% versus 0.68%, OR 11.34, *p* < 0.001 for the Humana registry base. They also reported that revisions performed within 90 days led to more infections than revisions performed within 1 year (11.76% versus 7.21%). Heckmann et al. and Schwarz et al. discovered a dose effect depending on the timing of revision after index surgery [10, 20]; the earlier the revision was performed, the higher the risk of infection, with an infection rate of 14.7% for revisions within the first month and 10.6% between 2 and 3 months. This effect decreased until 12 months after the index surgery.

The study found no infections associated with immediate revision. A possible explanation for the favorable risk–benefit ratio of immediate revision is the existence of a window of opportunity for earlier revision. However, quick decision-making is crucial. Further analysis of the revision procedures revealed no new cases of intubation, only a few instances of antibiotic administration, and a very short operative time. In most cases, only the femoral head was replaced. Revision joint arthroplasty implant costs accounted for more than 50% of the total hospital costs, as compared with 43% for primary procedures. The total hospital costs for revision cases increased by 161% [19]. The length of stay for immediately revised THA was similar to that of primary THA in our series [30].

Making a decision for immediate revision is challenging and sometimes subjective. The decision should consider several procedure-related parameters, such as the type and severity of the anomaly, the patient’s contralateral side to be operated on in the short or medium term to correct the LLD, the patient’s comorbidity, and the tolerance of the first procedure. Additionally, environmental factors, such as operative time, additional operative time, late end of the procedure, available teams, and operating rooms should also be considered. While the event is rare, the psychological impact on the surgeon and their team is significant, and the indications for immediate revision are not codified. It is essential to note that immediate revision does not increase the infection rate and can help surgeons make informed decisions.

The study had some limitations. First, it was a retrospective study, although the data were collected prospectively. However, as no patients were lost to follow-up in the immediate revision group, the retrospective model did not affect the results. Second, the results may not be reproducible because all surgeons were highly experienced in an institution where a team performs > 1400 THAs per year. Third, although the sample size was small, the number of subjects exceeded the required amount, a 2 year minimum follow-up period was completed, and there were no lost-to-follow-up cases to report. Fourth, the acknowledged risk factors for PJI, such as intraoperative blood loss, previous use of immunosuppressants, and preoperative hypoproteinemia were not examined [31]. Finally, the major limitation was the timing of postoperative radiography. In our institution, a dedicated team performs radiography in the operating room, and surgeons can view the radiographs before the patient leaves the recovery room. However, in several institutions, postoperative radiography is performed at a later onset, sometimes the day after surgery, making immediate revision impossible. Although clinically suspected, only the radiograph can confirm and explain a postoperative LLD (implant positioning, size, and comparison with preoperative templating).

The study has several strengths, including its design and observation of a well-defined population (patients with primary THA undergoing immediate revision). The minimum follow-up period was 2 years, ensuring that the infection rate was not underestimated. To our knowledge, this is one of the few studies that account for the immediate revision of imperfections after primary THA. In a well-trained center with experienced joint surgeons, immediate revision appears to be a safe option when imperfections, such as LLD or instability, are identified during postoperative bed transfer or on the immediate postoperative radiograph. Although studies have shown that early revision before 12 months significantly increases infection rates, immediate revisions do not appear to be associated with this burden. Immediate same-day revision is only an extension of the initial surgery.

## Conclusions

Immediate revision for early complications such as dislocation or LLD following primary THA appears to be a safe option and is not associated with an increased risk of PJI within 2 years. Surgeons should highly consider immediate revision to prevent poorer functional outcomes in the early postoperative complications if revision is not performed.

## Data Availability

The data supporting our findings can be obtained from the lead author.
